# Calcium deficiency and its implications for cardiovascular disease and cancer: Strategies for resolution via agronomic fortification

**DOI:** 10.1002/fsn3.4464

**Published:** 2024-09-23

**Authors:** Liping Cheng, Jiapan Lian, Yongfeng Ding, Xin Wang, Mehr Ahmed Mujtaba Munir, Shafqat Ullah, Erjiang Wang, Zhenli He, Xiaoe Yang

**Affiliations:** ^1^ Ministry of Education (MOE) Key Laboratory of Environmental Remediation and Ecosystem Health, College of Environmental and Resources Sciences Zhejiang University Hangzhou People's Republic of China; ^2^ Department of Medical Oncology, the First Affiliated Hospital Zhejiang University School of Medicine Hangzhou People's Republic of China; ^3^ The State Key Laboratory of Chemical Engineering, Department of Chemical Engineering Tsinghua University Beijing People's Republic of China; ^4^ State Key Laboratory of Food Science and Technology Jiangnan University Wuxi People's Republic of China; ^5^ Indian River Research and Education Center, Institute of Food and Agricultural Sciences University of Florida Fort Pierce Florida USA

**Keywords:** agronomic biofortification, calcium intake, cancer, cardiovascular disease (CVD), human health, plant‐based dietary calcium density

## Abstract

Calcium (Ca) is a vital nutrient essential for structural development and signal transmission in both plants and animals. In humans, inadequate calcium intake has been correlated with various diseases, including osteoporosis, cardiovascular and cerebrovascular diseases, and cancer. In areas where plants serve as a main dietary source, calcium intake is significantly lower than the recommended adequate intake, notably in low‐ and middle‐income countries (LMICs). Exploring the connections between calcium consumption and cardiovascular disease (CVD) and cancer has significant implications for public health, given that these two conditions are the primary contributors to global mortality. This study conducted a systematic review of existing literature to assess the evidence regarding calcium intake and its effect on blood pressure control, stroke prevention, and the controversial association with myocardial infarction. Furthermore, the preventive effect of calcium intake on tumor development, particularly in cancer prevention, was discussed. The study explores the potential of agronomic biofortification as a key strategy to enhance plant‐based dietary calcium density and improve human health. By advocating for the incorporation of calcium‐rich plants and plant‐derived products, alongside appropriate calcium supplementation, the study emphasizes the economic and practical benefits of plants as a calcium source. This approach is aligned with global dietary patterns and socioeconomic disparities. The review also highlights the need for further research to better understand the mechanisms through which agronomic biofortification can increase dietary calcium intake and reduce the risks of CVD and cancer associated with calcium deficiency. Ultimately, this study aims to deepen our understanding of the complex relationship between calcium intake and health.

## Graphical abstract

Epidemiological evidence has shown that calcium deficiency is linked to increased risks of cardiovascular disease and cancer. As a result, current dietary guidelines recommend adequate calcium supplementation to help prevent these conditions. Moreover, agronomic fortification offers an innovative and sustainable approach to improving dietary calcium intake by enhancing both the calcium content and its bioavailability.

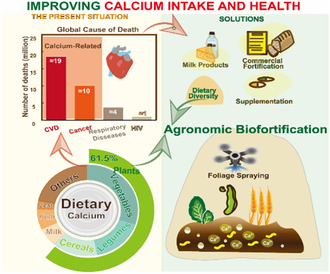

## 1 INTRODUCTION

Calcium, an ample mineral in human bodies, plays pivotal roles in various biological processes, encompassing both physiological and pathological aspects (Cormick & Belizán, 2019). Its metabolism and homeostasis are intricately regulated by hormones and vitamin D (Fleet, 2017; Peacock, 2010). Calcium is important to maintain teeth and bones (99% of calcium is stored in bones and teeth), muscle contraction, and other functions, such as vascular tone and electrical signaling (Cormick & Belizán, 2019; Peacock, 2010).

CVD and cancer stand as two major global causes of morbidity and mortality (Roth et al., 2020; Sung et al., 2021; Virani et al., 2021). Identifying modifiable risk factors is crucial for intervening in preventing these diseases, holding significant public health implications (Koene et al., 2016; Roth et al., 2020). Epidemiological research and precisely organized laboratory tests jointly indicate the possible influence of calcium consumption on CVD and cancer risks (Michaëlsson et al., 2013; Park et al., 2009). Evidence regarding the relationships between calcium intake and diseases such as CVD (Li et al., 2012; Zhu et al., 2021) and cancer (Abbas et al., 2013; Kesse‐Guyot et al., 2007; Zhang et al., 2011) remains inconclusive.

Serving as a fundamental nutritional cornerstone, calcium is integral to structural integrity and signal transmission in animals and plants (Thor, 2019). Diseases stemming from calcium deficiency underscore the critical importance of ensuring sufficient calcium intake across all age groups, whether obtained directly from food sources or through supplements. In the developing countries, the dietary landscape differs from the developed nations, relying heavily on plant‐based sources like grains, vegetables, and legumes, as opposed to dairy products dominating calcium intake (Lotfi et al., 2023). Unfortunately, calcium intake in most of the developed and developing countries often falls below the recommended daily calcium intake, resulting in widespread health challenges (Dayod et al., 2010). Appropriate calcium intake yields numerous health benefits, such as reduced blood pressure (especially in young individuals), osteoporosis prevention, and lower cholesterol levels (Daley & Myrie, 2021). Therefore, ensuring adequate calcium intake in the diet is essential for maintaining health.

In many countries, especially in developing nations, plant‐based diets are a major source of food. Agronomic biofortification and food fortification strategies are effective methods to increase calcium content in plant‐based diets, thereby enhancing the levels of dietary calcium (Ofori et al., 2022). Research has demonstrated the efficacy of agronomic biofortification techniques, such as nutrient enhancement, soil application, and foliar application, in enhancing the calcium content of staple crops and plant‐derived products, offering a sustainable solution to calcium deficiency (Chaudhary et al., 2022; White & Broadley, 2009). The absorption of calcium nutrition is often dependent on both the calcium content itself and the bioavailability of calcium (Castro‐Alba et al., 2019). Dairy products are rich in calcium nutrition and have high bioavailability, whereas the bioavailability of calcium in staples, soybeans, fruits, and vegetables is generally low. Even if vegetables have a high calcium nutrition content, the presence of high levels of phytic acid and oxalic acid in plants can inhibit calcium absorption. Therefore, similar to the absorption of other mineral nutrients, the key to enhancing calcium absorption lies in increasing the absorption, transport, and/or accumulation of the edible parts of plants (Gustiar et al., 2020; Muleya et al., 2024; Pessoa et al., 2021; White & Broadley, 2009). Agronomic biofortification techniques not only increase the bioavailability of calcium but also contribute to the overall nutritional quality of food. Additionally, food fortification strategies involve adding calcium to foods during processing or production to increase their calcium content. Common calcium‐fortified foods include yogurt, breakfast cereals, bread, juice, tofu, and some other dairy products. However, for most of the world's population, especially those in LMICs, the actual accessibility and practicality are limited. This study aims to provide an overview of calcium homeostasis mechanisms and recommended intake, systematically investigating the correlation between calcium intake and the incidence of CVD and various cancers. The implications of this comprehensive review have the potential to shape recommendations and guide evidence‐based health policy decisions. It is noteworthy that biofortification of crops represents an economically viable and feasible intervention within the agricultural production process, offering one of the most cost‐effective measures to alleviate malnutrition prevalent in numerous countries. The enhancement of calcium nutrient absorption is not solely contingent upon increased calcium content but also correlates with improvements in calcium bioavailability. Consequently, agronomic biofortification methods for calcium nutrition occupy a crucial position in augmenting calcium absorption and mitigating the risk of CVD and various cancers attributable to calcium nutrition imbalances.

## 2 CALCIUM HOMEOSTASIS AND REQUIREMENT

### 2.1 Major mechanisms of calcium homeostasis

**FIGURE 1:**
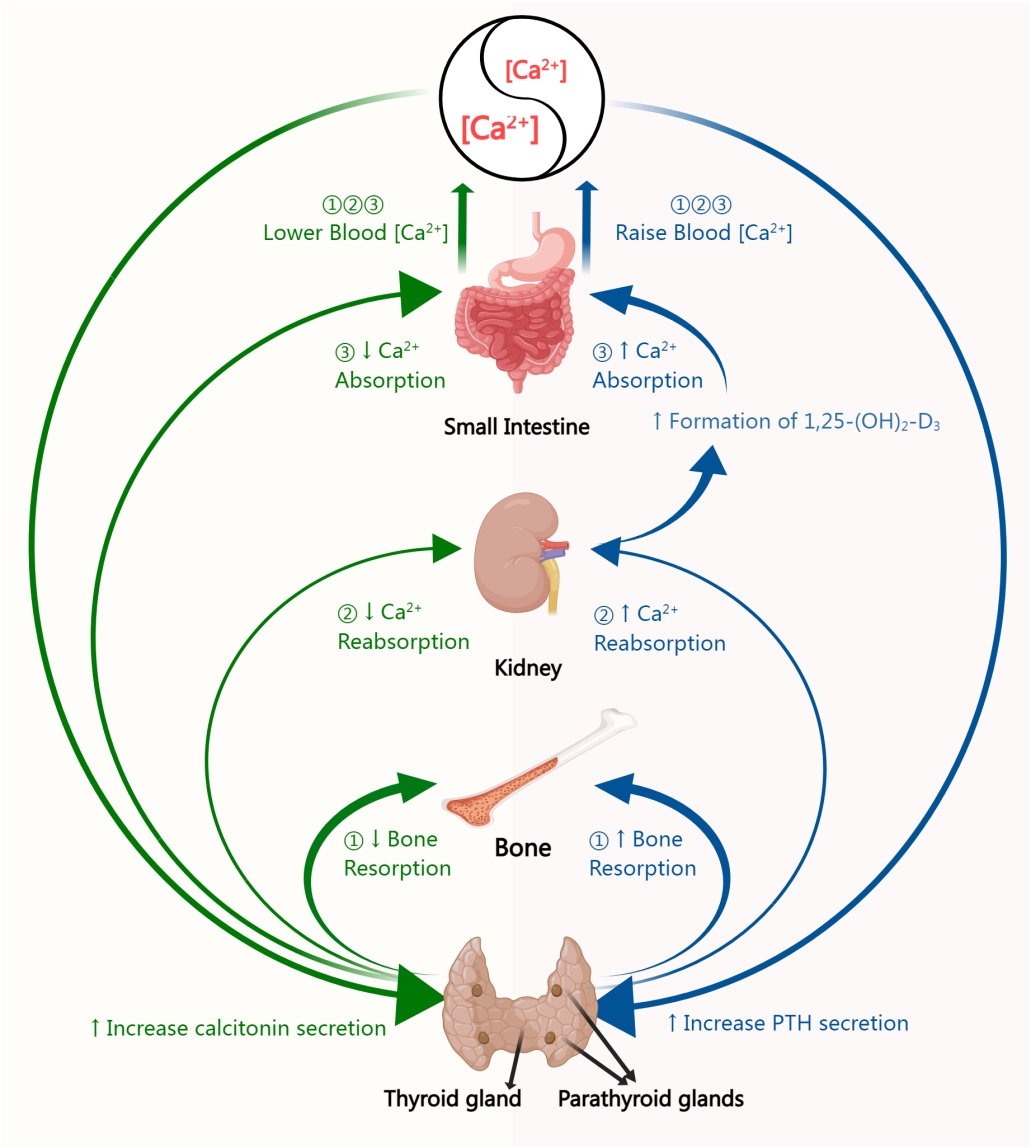
The main mechanism flowchart of calcium homeostasis.

Considering the essential role of calcium, maintaining the homeostasis of calcium levels in the body is extremely important (Matikainen et al., 2021). As shown in Figure 1, when blood calcium levels decrease, as seen in cases of inadequate calcium intake, the parathyroid glands detect this change and respond by secreting parathyroid hormone (PTH). It stimulates the transformation of vitamin D into its activated structure, 1,25‐dihydroxyvitamin D3, within the kidneys. The effective structure, working in conjunction with PTH, serves to minimize calcium loss in urine and promote calcium release from the bones. Additionally, effective vitamin D increases the uptake of calcium nutrition throughout the gastrointestinal system. Once bloodstream calcium concentrations are normal, all of these mechanisms of increasing calcium are turned off (Peacock, 2010). However, the thyroid gland detects higher blood calcium levels, which triggers the release of calcitonin. On the contrary, calcitonin subsequently inhibits the secretion of PTH, decreases both the absorption of calcium in the intestines and bone resorption, and increases calcium excretion in urine, resulting in the downregulation of the level of calcium and maintaining homeostasis.

### 2.2 Calcium intake requirement and dietary sources of calcium

**FIGURE 2:**
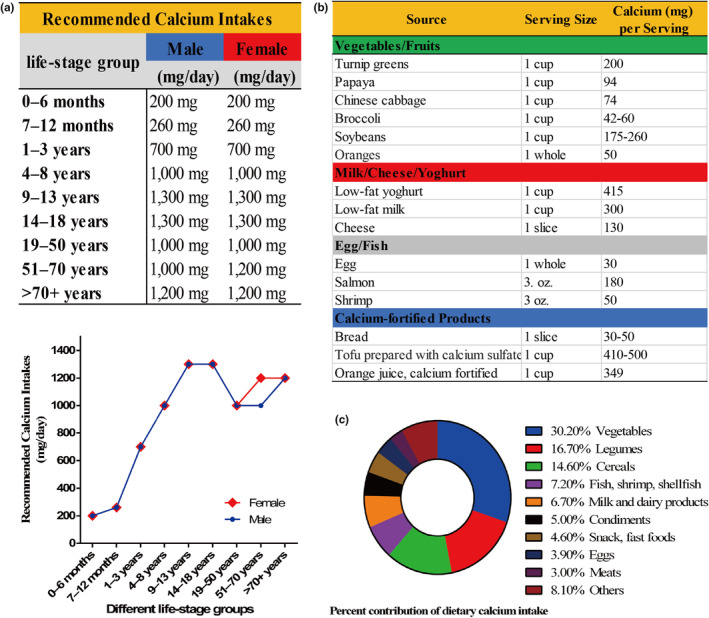
The overview of recommended calcium intakes and common dietary sources of calcium. (a) Chart and line graph showing the recommended calcium intakes for males and females across different age groups; (b) Illustration of the calcium content in various dietary sources; (c) Pie chart representing the composition of the human diet, specifically the proportions of foods that typically contribute to calcium intake.

According to Figure 2a, the recommended dietary allowances (RDA) for calcium vary between 1000 mg and 1300 mg for individuals over 4 years of age, contingent upon factors such as age, sex, and pregnancy (Cormick & Belizán, 2019; Ross et al., 2011). Specifically, the calcium intake for children within the 9–13 age range and teenagers in the 14–18 age group is recommended at 1300 mg per day. For adults, the suggested calcium assimilation is 1000 mg every 24 hours, with an increase to 1200 mg among women aged 50 years and older and men aged 70 years and beyond. Caution is advised regarding potential side effects, such as hypercalcemia, vascular calcification, and nephrolithiasis, leading to a general recommendation that calcium supplementation should not exceed 2000 mg per day (Ross et al., 2011).

Calcium, a naturally abundant mineral, is present in plant food (Weaver & Peacock, 2019), which is recognized as a beneficial source for maintaining normal physiological functions. Noteworthy foods with high levels of calcium encompass green vegetables (such as broccoli and watercress), legumes (such as broad beans), milk‐based foods (including cheese and yogurt), fish, and others (Cormick & Belizán, 2019; Plantz & Bittar, 2023). Figure 2b shows the calcium content in representative foods. A study by Huang et al. gathered dietary‐related information from over 11,000 Chinese adults aged across 15 different areas, highlighting the percentage contributions of various food groups to dietary calcium intake (Huang et al., 2018) (Figure 2c). Notably, vegetables emerged as the primary sources, constituting 30.2% of the dietary intake, followed by legumes at 16.7% and cereals at 14.6%. And for other groups, each contributed no more than 10.0% to the overall dietary calcium consumption.

### 2.3 Inadequate calcium intake and global health burden

The recognition of modifiable risk factors is crucial for preventing diseases with suboptimal prognoses, such as CVD and cancer. Balk et al. analyzed the calcium intake among populations across more than 70 countries with available data (Balk et al., 2017). The dietary calcium intake in one day extends from 175 mg in Nepal to 1233 mg in Iceland. Notably, daily calcium intake falls well below recommended levels in most developing countries (Balk et al., 2017; Kumssa et al., 2015). This inadequacy in calcium intake extends to special groups such as adolescents, pregnant women, and older adults in developed countries (Balk et al., 2017; Cormick et al., 2019; Kumssa et al., 2015; Zhao et al., 2017). In addition, Vatanparast et al. analyzed data from two studies respectively conducted in 2004 and 2015 in the Canadian population, revealing a decrease in calcium absorption from two major sources (diet and supplemental sources) (Vatanparast et al., 2020). The widespread population with insufficient calcium intake poses a significant challenge to global public health. Addressing this issue has become an urgent priority, emphasizing the need for comprehensive strategies to enhance calcium intake across diverse populations.

## 3 CALCIUM INTAKE AND CARDIOVASCULAR DISEASE

### 3.1 Calcium intake and hypertension

Typically, if the systolic blood pressure exceeds 140 mmHg and/or the diastolic blood pressure falls below 90 mmHg, it is categorized as hypertension. This condition, often stemming from a combination of environmental and genetic factors, is widely acknowledged in the medical community as a primary contributor to CVD (2018) (Carey et al., 2022; Mills et al., 2020). It represents a substantial public health burden, with over 30% of global adults grappling with hypertension (Mills et al., 2020).

Cormick et al. incorporated data from 18 randomized controlled trials involving 3140 participants and indicated that increased calcium intake has a subtle yet meaningful influence in lowering blood pressure in normotensive individuals, suggesting a potential role in hypertension prevention (Cormick et al., 2022). In another meta‐review of prospective investigations, Jayedi et al. observed a correlation of 0.89 between higher calcium intake and a reduced risk of hypertension. (Jayedi & Zargar, 2019). Furthermore, in the case of adolescents, reinforcing dietary calcium nutrition has been correlated with a reduced likelihood of hypertension or prehypertension (Kajale et al., 2016; Quaresma et al., 2019). Simultaneously, for pregnant women during mid‐pregnancy, maintaining consistently low levels of calcium intake has shown a strong association with the development of post‐pregnancy hypertension (Egeland et al., 2017).

The relationship between calcium intake and blood pressure involves mechanisms such as influencing intracellular calcium and vascular volume (Villa‐Etchegoyen et al., 2019). Insufficient calcium intake results in increased levels of PTH and calcitriol, elevating intracellular calcium relating to the smooth muscle cells in vascular tissue and inducing vasoconstriction (Morfis et al., 1997; Sadideen & Swaminathan, 2004; Villa‐Etchegoyen et al., 2019). Additionally, low blood calcium levels (such as insufficient calcium absorption), along with PTH, can trigger the secretion of renin, leading to the synthesis of angiotensin II and aldosterone, ultimately affecting vascular volume (Villa‐Etchegoyen et al., 2019; Yuasa et al., 1996).

### 3.2 Calcium intake and stroke

Wang et al. carried through a meta‐analysis including 18 investigations and concluded that calcium intake showed a positive role in preventing stroke, with a relative risk (RR) of 0.89 and a 95% confidence interval (CI) of 0.81–0.97. Notably, this protective role was more pronounced in the Asian region (Wang et al., 2023). Zhu et al. undertook research involving 6411 participants who were stroke‐free at baseline and throughout the observation period covering 32 thousand person‐years, and finally, 179 examples were reported experiencing stroke. Based on the above analysis, it was found that there is a noteworthy relationship between elevated calcium absorption and a reduced risk of stroke, with a Ratio of Hazards (RoH) of 0.53. Further analyses revealed that calcium intake had a pronounced inverse connection to stroke risk among men, while no relevant correlation was noticed among women (Zhu et al., 2021).

### 3.3 Calcium intake and myocardial infarction

Li et al. conveyed that the absorption sources of calcium from different pathways, including dietary sources and supplementation, exhibited certain connections to self‐reported myocardial infarction (MI). This research indicates that calcium derived from supplementation sources may contribute to an elevated risk of MI (Li et al., 2012). However, this conclusion remains a subject of considerable controversy (Guessous & Bochud, 2013; Lewis et al., 2012; Prince et al., 2012). Lewis et al. contended that the increase in patient self‐reported MI in individuals receiving calcium supplementation may be partly attributed to a rise in gastrointestinal events inaccurately perceived by the patient (Lewis et al., 2012). Additionally, Lewis and colleagues advocated that the analysis should have included unstable angina, given its similarities to MI (Prince et al., 2012). The contention put forth is that organic calcium sources, specifically dietary calcium intake, do not cause an increase in MI risk and should be prioritized in recommendations (Li et al., 2012).

### 3.4 The Summary of calcium intake and cardiovascular disease

In a comprehensive meta‐analysis encompassing 35 inquiries conducted by Naghshi et al., they observed that calcium absorption exhibited a contrary correlation with overall mortality (Naghshi et al., 2022). Conflicting evidence regarding the cardiovascular risks associated with calcium intake exists (Kong et al., 2017; Myung et al., 2021). However, upon reviewing the available evidence, it becomes evident that adequate calcium intake is bound to a lowered threat of hypertension and stroke. The debate revolves around whether more calcium intake is correlated with an elevated risk of MI. Notably, hypertension is reported as a major risk factor correlated with several CVDs, including stroke, as well as cancer in humans (Hibler & Lloyd‐Jones, 2021; Lu et al., 2019; Mills et al., 2020). The remarkable link between adequate calcium intake and a decreased risk of hypertension also provides indirect evidence for the effective prevention of the occurrence of stroke. Considering that gender is highly relevant to myocardial infarction (Benjamin et al., 2017; Dreyer et al., 2015), future investigations exploring the relationship between MI and calcium intake should consider stratification by gender or include gender as a confounding factor in the model.

There is a concern that the different sources of calcium intake might have different roles in the association of CVD. Yang et al. performed a meta‐analysis to analyze the associations between CVD risks and calcium from dietary intake or supplemental intake (Yang et al., 2020). They found that calcium intake from calcium supplements might raise the risk of MI, while dietary calcium intake does not enhance the risk of CVD. Atherosclerosis, the dominant pathological basis for the occurrence and development of CVD (Hansson, 2005), is highlighted in a study by Anderson et al., which suggested reduced risks of atherosclerosis if dietary calcium is obtained from food and refraining from calcium supplementation (Anderson et al., 2016). This underscores the protective role of calcium intake from food sources on the cardiovascular system. Takata et al. also validated that calcium intake from dairy food sources was connected to a lower risk, rather than exogenous calcium supplementation (Takata et al., 2013). Interestingly, the association between calcium intake and the risk of CRC seemed unaffected by intake source (Keum et al., 2014; Zhang et al., 2016).

## 4 CALCIUM INTAKE AND THE RISK OF CANCER

Calcium metabolism is intricately regulated through three primary pathways (Weaver & Peacock, 2019). Intestinal absorption, being the crucial and initial phase of calcium metabolism, is typically considered to have two sources: dietary food and calcium supplements. Thus far, the relationship between calcium intake and the incidence of cancer demonstrates variability across different types of cancer.

### 4.1 Digestive system tumors

Heilbrun et al. performed a nested case–control study in Hawaiian Japanese men to examine the association between calcium intake and risk of colon cancer and found that there is no significant association (Heilbrun et al., 1986). Conversely, Kim et al. observed a decreased risk of colorectal cancer (CRC) with higher calcium intake in a cohort of young women (Kim et al., 2022). A meta‐analysis including 24,353 CRC suggested a protective effect, indicating a decrease in CRC risk for elevated daily calcium intake (Lopez‐Caleya et al., 2022). In a case–control study by Fan et al. on pancreatic cancer (pancreatic cancer, *n* = 150; control, *n* = 459) in Minnesota, no significant association between calcium intake and pancreatic cancer risk was found (Fan et al., 2021). However, Hoyt et al. reported a negative association between total calcium intake and pancreatic cancer risk in a study involving 58,477 participants (HR = 0.67, *p* < .05) (Hoyt et al., 2021). Shah et al. performed a prospective cohort analysis (*N* = 536,403) to assess the relationship between dietary and supplemental calcium intake and the incidence of gastric cancer. They found that elevated calcium intake was related to a decreased risk of gastric cancer (*p* = .05) (Shah et al., 2020). Moreover, Li et al. conducted a meta‐analysis (close to 3.4 thousand cases and close to 350 thousand controls) to assess the link between calcium absorption and the occurrence of esophageal malignancy (Li et al., 2017). Their findings suggest that higher calcium intake might reduce the risk of esophageal cancer, particularly for esophageal squamous cell cancer.

### 4.2 Tumors in women

The current evidence on the relationship between calcium intake and breast cancer risk is inconclusive. Some studies suggest a protective effect of high dietary calcium intake against breast cancer risk (Boyapati et al., 2003; Kesse‐Guyot et al., 2007; Zhang et al., 2011). However, in a large European prospective cohort study, Abbas et al. found no association between dietary calcium intake or vitamin D and the risk of breast cancer (Abbas et al., 2013). Similarly, a study by Wu et al. (2021), pooling individual‐level data from over 1 million women, indicated that dairy or calcium intake is unlikely to be linked with the risk of breast cancer.

The heterogeneity in results is further highlighted by research from Kawase et al., suggesting that the effects of calcium intake against the incidence of breast cancer may vary by menopausal status and receptor status (Kawase et al., 2010). In another prospective study involving over ten thousand women, it was shown that moderate calcium absorption could potentially lower the danger of breast cancer among only postmenopausal women (Fernandez‐Lazaro et al., 2021). In Swedish populations, Larsson et al. found no overall association between calcium intake and breast cancer risk, but a significant decrease was found for ER‐negative/PR‐negative (ER−/PR−) breast cancer (*p* = .02) (Larsson et al., 2009).

Ovarian cancer is a prevalent type of cancer in females, with approximately 75% of cases diagnosed at an advanced stage with poor survival (Lee et al., 2018; Lheureux et al., 2019). Calcium intake is potentially affiliated with the hazard of ovarian cancer (McCarty, 2000; Toriola et al., 2010). Nevertheless, findings vary across different studies. (Bidoli et al., 2001; Faber et al., 2012; Koralek et al., 2006; Qin et al., 2016). In a meta‐analysis conducted by Xu et al. involving nearly 980 thousand participants, the findings suggested that both dietary calcium intake and supplementary calcium intake were correlated with a decreased health threat of ovarian cancer (Xu et al., 2021). Similarly, another meta‐analysis by Liao et al., encompassing twenty‐nine case–control or cohort studies, indicated that higher dietary calcium intake was linked to a decreased risk of ovarian cancer (RR = 0.71, 95% CI 0.60, 0.84) (Liao et al., 2020).

### 4.3 Lung cancer

The association between calcium intake and lung cancer risk has been investigated in several studies with inconsistent results. Takata et al. conducted a population‐based, prospective cohort study and revealed that calcium intake is protective for lung cancer, with the highest quintile versus the lowest quintile showing a protective effect (0.66, 95% CI 0.48, 0.91) (Takata et al., 2013). In contrast, Mahabir et al. observed no significant associations between total calcium intake (diet + supplement) and lung cancer risk in their study (Mahabir et al., 2010). Zhou et al., in a case–control study, reported an adjusted odds ratio (OR) of 1.64 (95% CI = 1.17–2.29) for the highest quintile versus the lowest quintile of dietary calcium intake from food sources (Zhou et al., 2005).

To further clarify the roles of calcium intake in lung cancer incidence, Sun et al. performed a systematic meta‐analysis (Sun et al., 2021). They found that separate intake of calcium and vitamin D did not show a protective role in lung cancer. However, the combination of calcium and vitamin D intake showed a significantly reduced risk of lung cancer.

### 4.4 Prostate cancer

The association between calcium intake and prostate cancer risk has generated varied findings in different studies. Giovannucci et al. (1998) and Chan et al. (2001) reported that an increase in prostate cancer risk was linked to elevated calcium intake. In contrast, Schuurman et al. (1999), Tavani et al. (2001), and Baron et al. (2005) found no significant association between calcium intake and prostate cancer risk. A meta‐analysis conducted by Rahmati et al. aimed to determine the association between calcium intake and the risk of prostate cancer (Rahmati et al., 2018). This analysis suggested that high calcium intake might be linked to a small increased risk of prostate cancer (RR = 1.15, 95% CI: 1.04–3.46).

### 4.5 Mechanism underlying the calcium intake and cancer risk

Calcium intake has been connected to a decreased risk of several cancers. However, the underlying mechanisms through which calcium intake may act on carcinogenesis are still unclear. In a prospective cohort study, Yang et al. used validated food frequency questionnaires (FFQs) to investigate dietary information at baseline or every 4 years. They assessed tumor CASR protein expression in over seven hundred incident colorectal cancer patients that developed among over 130,000 individuals (Yang et al., 2018). They found that elevated calcium intake was correlated with reduced risk of CASR‐positive CRC but not CASR‐negative CRC, indicating a potential role for CASR in this context. In addition, in another study, the authors also assessed the densities of tumor‐infiltrating T‐cell subsets using IHC (first in 736 colorectal cancer cases and then developed among 136,249 individuals) (Yang et al., 2019). They found that calcium intake was correlated with the densities of tumor‐infiltrating T cell subsets, suggesting a possible role of calcium intake in cancer immunoprevention via modulation of T cell function. The regulation of insulin‐like growth factor‐1 (IGF‐1) by circulating PTH has been linked to cell proliferation and the inhibition of apoptosis (Khandwala et al., 2000). Coppola et al. found that overexpression of the IGF‐1 receptor induces malignant transformation of ovarian epithelial cells (Coppola et al., 1999). Thus, higher calcium intake may be negatively related to ovarian cancer risk via the downregulation of PTH and a series of subsequent molecular changes (Coxam et al., 1992).

### 4.6 Summary of calcium intake and cancer risk

Cancer represents a diverse set of complex human genetic diseases, and this current study explores the impact of calcium intake on different tumor types. Our findings suggest that the effect of calcium intake on cancer incidence varies across different cancer types. Previous studies have indicated that higher calcium intake tends to reduce the risk of CRC (Kim et al., 2022; Lopez‐Caleya et al., 2022; Zhang et al., 2016, 2020), gastric cancer (Shah et al., 2020), esophageal cancer (Li et al., 2017), and ovarian cancer (Liao et al., 2020; Xu et al., 2021). The association between calcium and the risk of CRC has been extensively explored. Allam et al. found that calcium supplements can bind heme in vitro and reduce fecal biomarkers in relation to the carcinogenesis of CRC (Allam et al., 2011). However, the association between calcium intake and lung cancer (Sun et al., 2021; Takata et al., 2023), pancreatic cancer (Fan et al., 2021; Hoyt et al., 2021), and breast cancer (Abbas et al., 2013; Kesse‐Guyot et al., 2007; Wu et al., 2021; Zhang et al., 2011) has been a subject of debate. Interestingly, researchers from different institutions found that higher calcium intake may be beneficial in reducing breast cancer risk for particular populations (Fernandez‐Lazaro et al., 2021) or subtypes (Larsson et al., 2009). It should be borne in mind that higher calcium intake increases the risk of prostate cancer (Rahmati et al., 2018). Despite the small increase in risk, caution is advised when administering calcium supplementation for men at high risk of prostate cancer.

Calcium deficiency remains a critical public health challenge, contributing to a range of health issues such as CVD, certain cancers, or osteoporosis. While traditional dietary recommendations and calcium supplementation have been key strategies in addressing this deficiency, these approaches often fall short in reaching underserved populations, particularly in regions with limited access to dairy products or where cultural dietary patterns do not emphasize calcium‐rich foods, such as in LMICs. This highlights the need for innovative strategies that can more effectively deliver adequate calcium through everyday diets, thereby reducing the burden of calcium‐related diseases globally.

Given the widespread prevalence of calcium deficiency and its significant impact on public health, it is essential to explore alternative and sustainable methods for increasing calcium intake at the population level. One promising approach lies in the enhancement of dietary calcium through plant‐based sources, particularly via agronomic biofortification. This strategy not only aligns with global efforts to improve nutrient intake through more accessible and culturally appropriate food sources but also addresses the socioeconomic disparities that often limit the effectiveness of conventional dietary interventions. The following section delves into the role of agronomic biofortification as a pivotal tool in this endeavor, discussing its potential to increase the calcium content of staple crops and its implications for public health.

## 5 FORTIFICATION OF PLANT‐SOURCE DIETARY CALCIUM

Agronomic biofortification primarily involves utilizing agronomic methods to enhance the bioavailability of macro‐ and micronutrients (such as minerals, calcium, zinc, iron, etc.) in staple food crops (Chaudhary et al., 2022). This process aims to increase the nutrient density/content of food crops while maintaining or improving yields and stress resistance and ensuring public acceptance in terms of food color and taste. It is considered a crucial approach to address nutrient deficiencies. In contrast to molecular and genetic approaches, agronomic biofortification necessitates existing crops and varieties to prioritize the enhancement of plant nutrient content (quality) while ensuring adequate calorie (yield). Agronomic biofortification is considered one of the most widespread and effective approaches for increasing crop nutrient levels. China, the United States, India, Russia, and Brazil, being major grain‐producing countries, have conducted extensive research in the field of agronomic biofortification. This underscores the significant relevance of agronomic biofortification in promoting crop nutrition, particularly for impoverished populations severely lacking in meat and dairy products (Chan et al., 2001; Schuurman et al., 1999). Calcium deficiency is one of the most prevalent conditions among nutrient deficiencies, and agronomic fortification stands out as a crucial tool for ameliorating symptoms of the connection with calcium deficiency.

### 5.1 Agronomic biofortification of calcium in plants

There is a concern that the different sources of calcium intake might have different roles in the association of CVD. Yang et al. performed a meta‐analysis to analyze the associations between CVD risks and calcium from dietary intake or supplemental intake (Yang et al., 2020). They found that calcium intake from calcium supplements might raise the risk of MI, while dietary calcium intake does not enhance the risk of CVD. Atherosclerosis, the dominant pathological basis for the occurrence and development of CVD (Hansson, 2005), is highlighted in a study by Anderson et al., which suggested reduced risks of atherosclerosis if dietary calcium is obtained from food and refraining from calcium supplementation (Anderson et al., 2016). This underscores the protective role of calcium intake from food sources on the cardiovascular system. Takata et al. also validated that calcium intake from dairy food sources was connected to a lower risk, rather than exogenous calcium supplementation (Takata et al., 2013). Interestingly, the association between calcium intake and the risk of CRC seemed unaffected by intake source (Keum et al., 2014; Zhang et al., 2016).

Agronomic biofortification employs various methods to increase the calcium content/density in the edible parts of crops. These methods include remediating soil properties (such as adjusting soil acidity and increasing organic matter content), application of plant‐available calcium fertilizers to the soil, foliar application of calcium, use of growth regulators, or microbial amendment (Baron et al., 2005; Rahmati et al., 2018; Tavani et al., 2001). These approaches have proven effective in enhancing the accumulation of calcium in crops.

Plants, including vegetables, legumes, and cereals, emerge as the primary sources of dietary calcium. According to FAO reports, the current global cereal production is projected to exceed 2.8 billion tons, reaching a historical peak. Wheat and rice stand out as the two most extensively demanded cereal crops worldwide. Nearly 50% of the global population relies on rice as a staple food, with rice consumption constituting three‐quarters of the daily dietary energy intake for this portion of the population (Baron et al., 2005; Tavani et al., 2001). Therefore, calcium fortification of cereals has proved to be one of the most economically effective approaches to impact populations suffering from calcium deficiency. Under existing commercial cultivation conditions and fertilizer exploration trials, it has been observed that many crops, particularly when supplied with calcium fertilizers in the form of nutrient solutions, accumulate calcium levels in their edible parts far exceeding the calcium levels required for their growth. Common types of calcium fertilizers include lime, gypsum, Ca(NO_3_)_2_, calcium phosphate, and calcium sulfate. For instance, lime not only elevates soil pH but also provides an ample source of calcium for the surface soil layer (Hamid et al., 2022). Broadley and White (2010) have demonstrated that fertilizing cereals for calcium fortification can achieve a nutrient increase goal of up to 50%, thereby meeting the recommended calcium intake for 300,000 individuals.

Moreover, soil fertilization itself possesses drawbacks such as low availability, slow nutrient conversion, and poor economic benefits. Coupled with the inherent specificity of calcium elements, which are prone to crystallization and challenging to transport, foliar and fruit application of soluble calcium fertilizers is also widely employed in horticultural crops to prevent calcium deficiency symptoms and enhance crop nutrition (Hou et al., 2022; Wang & White, 2022). The application of calcium is efficient when foliar spraying and nanofertilizers are employed (Carmona et al., 2022). Coelho et al. conducted a study where, approximately every 9 days, four foliar sprays were applied to potato plants of the Agria and Ross varieties using a solution of 6 kg HA^−1^ CaCl_2_ or 4 kg HA^−1^ Ca(NO_3_)_2_ per application. The results revealed that both fertilizers increased the accumulation of calcium in the edible parts of potatoes, particularly concentrated in the central tissue around the equator (Coelho et al., 2022). Research conducted by Ranjbar et al. indicates that foliar application of nano‐calcium and calcium chloride can enhance the quality of apples, delaying maturation and senescence, thereby facilitating storage and transportation and reducing the economic costs related to apples. However, no report on the changes in calcium content was provided (Ranjbar et al., 2018). Pessoa et al. conducted two exogenous calcium foliar sprays during the development of Rocha pear fruits. Upon harvesting, higher calcium content was observed in the epidermis and central region of the fruit in all the foliar spray treatments (Pessoa et al., 2021). Fitra Gustiar et al. fertilized mustard (*Brassica juncea* L.) and lettuce (*Lactuca sativa*) in a hydroponic system with varying concentrations of calcium. The results indicate that the 300 ppm Ca treatment not only significantly increased leaf number and leaf greenness levels but also increased plant calcium content (Gustiar et al., 2020). In the long term, agronomic biofortification measures on vegetables, fruits, grains, and other plants, without altering dietary habits, exhibited greater feasibility compared to alternative approaches. This shift allows a transition from merely preventing diseases caused by calcium deficiencies to ensuring individuals attain sufficient intake.

### 5.2 Staple foods fortified with calcium

While grains constitute a primary dietary component in most low‐ to middle‐income countries worldwide, the calcium content in grains is not notably advantageous without additional calcium fortification. Additionally, calcium‐rich outer layers such as bran and husk are often removed during the refining process. The United Kingdom is the only country that legally mandates the addition of nutritional elements such as calcium and iron to processed wheat flour. The calcium content is required to fall within the range of 235–390 mg/100 g. Cormick et al. modeled calcium fortification data from seven countries using dietary intake databases. The simulation results suggest that flour containing 156 mg/100 g of calcium could reduce the incidence of diseases tied to low calcium intake, with this standard being referenced from the United Kingdom (Cormick et al., 2021). Muleya et al. assessed the total calcium supply and bioavailable calcium supply levels in 25 plant‐based products. They identified kale, millet, and white bread as potentially superior sources of plant‐based calcium. It was noted that white bread, rich in calcium carbonate, could be an ideal calcium source in staple foods. The study advocates for large‐scale fortification of staple grains as a feasible approach to enhance dietary calcium intake, especially for vulnerable populations (Muleya et al., 2024). Furthermore, in regions deficient in meat and dairy products, the supplementation of calcium in grains through vegetables and fruits, including kale, and the formulation of mixed beverages combining grains with fruits and vegetables are considered economically feasible for effectively reducing calcium deficiency (Broadley & White, 2010). Considering the low calcium intake in the populations of low‐ to middle‐income countries and their predominant reliance on plant‐based dietary sources, the external addition of calcium to processed cereal grains emerges as a potentially feasible strategy to address the widespread issue of low calcium intake in these regions.

However, there are several challenges linked to the food fortification policy of externally adding calcium to flour or cereal juices. A crucial aspect is the variation in the levels and standards of food fortification policies across different countries. Additionally, the insufficient depth and clarity of scientific research on the bioavailability of calcium‐fortified foods, especially those involving flour or cereal juices, can result in limited effectiveness. Excessive external calcium addition or the overconsumption of calcium‐fortified flour or juices may negatively impact health, contradicting the intended benefits of calcium fortification. Moreover, the success of grain fortification measures requires substantial support not only at the individual level but also from national governments, research institutions, businesses, and other societal organizations (Olson et al., 2021). Therefore, the external addition of calcium to processed grains has inherent limitations.

## 6 SUMMARY AND RECOMMENDATION FOR FUTURE RESEARCH

As previously mentioned, CVD and cancer, being major causes of mortality globally, have also been linked to calcium intake. Currently, guidelines extensively recommend calcium intake of 1000 to 1200 mg, preferably from the diet, with supplements being appropriate in patients who were unable to achieve adequate intake from dietary sources (Zarzour et al., 2023). As recommended by Plantz et al., sufficient calcium intake is achievable from a well‐balanced diet with multiple alternative natural calcium sources, including vegetables (e.g., broccoli, kale), dairy products (e.g., milk, yogurt, cheese), and foods rich in calcium (e.g., fruit juices and some grains) (Plantz & Bittar, 2023). However, it is worth noting that in developing or low‐income countries, their dietary sources are mainly grains, vegetables, or legumes, and a plant‐based diet becomes the main pathway for their calcium intake. Therefore, consuming calcium‐rich plants has become the most effective and primary way to solve diseases caused by calcium deficiency in this group of people. Initiatives aimed at augmenting plant calcium content through strategies like crop nutrient fortification, technological innovations in processing, and policy interventions become critical. These endeavors are essential not only for addressing calcium deficiency but also for fortifying our body's defense system against the emergence of diseases correlated with inadequate calcium levels (Bourassa et al., 2022; Park et al., 2011; Theobald, 2005).

## 7 CONCLUSION

The large population with insufficient calcium intake poses significant pressure on global public health. Attaining recommended calcium intake offers substantial health benefits, particularly in preventing CVD like hypertension, stroke, and various cancers, including colorectal cancer. Major mechanisms of calcium homeostasis indicate a substantial correlation between calcium absorption and human health. To mitigate potential side effects, emphasis should be placed on obtaining calcium from dietary sources, and calcium supplementation is recommended when necessary to ensure sufficient calcium intake. Without modifying human dietary habits, employing agronomic biofortification on crops like grains, vegetables, fruits, and soybeans optimally utilizes existing crop and variety advantages, enhancing crop calcium nutrition. This approach is expected to effectively tackle human calcium deficiency, particularly in low‐ to middle‐income countries (LMICs) that depend on plants, positioning itself as an inevitable choice within the global context of dietary patterns and socio‐economic disparities.

## AUTHOR CONTRIBUTIONS

**Liping Cheng:** Data curation (equal); formal analysis (equal); investigation (equal); methodology (equal); validation (equal); visualization (equal); writing – original draft (equal); writing – review and editing (equal). **Jiapan Lian:** Conceptualization (equal); methodology (equal); writing – review and editing (equal). **Yongfeng Ding:** Investigation (equal); methodology (equal); writing – review and editing (equal). **Xin Wang:** Investigation (equal); methodology (equal). **Mehr Ahmed Mujtaba Munir:** Formal analysis (equal); writing – review and editing (equal). **Shafqat Ullah:** Methodology (equal); validation (equal). **Erjiang Wang:** Methodology (equal); visualization (equal). **Zhenli He:** Formal analysis (equal); supervision (supporting); writing – review and editing (equal). **Xiaoe Yang:** Data curation (equal); funding acquisition (lead); project administration (lead); resources (equal); supervision (lead); writing – review and editing (equal).

## FUNDING INFORMATION

The authors acknowledge the financial support from the Department of Science and Technology of Zhejiang Province (#2023C02002).

## CONFLICT OF INTEREST STATEMENT

The authors declared no conflicts of interest.

## Data Availability

Data sharing is not applicable to this article as no datasets were generated or analyzed during the current study.
